# Effect of Simulated Soft-Tissue Edema on Ultrasonographic Detection of Nondisplaced Dentoalveolar Fractures: An Ex Vivo Animal Model Study

**DOI:** 10.3390/tomography12100150

**Published:** 2026-10-08

**Authors:** Sedef Ayşe Taşyapan, Murat Mert Atapek, Elif Yıldızer

**Affiliations:** 1 Faculty of Dentistry Oral and Maxillofacial Radiology Department, Istanbul University, 34116 Istanbul, Türkiye; 2 Faculty of Dentistry Oral and Maxillofacial Radiology Department, Yeditepe University, 34728 Istanbul, Türkiye

**Keywords:** ultrasonography, dentoalveolar fracture, soft-tissue edema, cone-beam computed tomography, maxillofacial trauma, diagnostic accuracy

## Abstract

**Objectives:** This single-blinded ex vivo diagnostic accuracy study evaluated high-frequency ultrasonography for detecting nondisplaced dentoalveolar fractures and assessed the effect of simulated post-traumatic soft-tissue edema. **Methods:** Eighteen fresh sheep heads provided 108 anatomically distinct sites (54 fractured and 54 non-fractured controls). Two blinded oral and maxillofacial radiologists independently assessed each site, yielding 216 observer-level evaluations. Cone-beam tomography (CBCT) served as the reference standard. Edema was simulated by submucosal injection of 0.5, 1.0, or 1.5 mL sterile saline. **Results:** Simulated edema was associated with significantly lower odds of correct ultrasonographic classification in the GEE analysis accounting for within-site clustering (OR = 0.217; 95% CI, 0.096–0.491; *p* < 0.001). Accuracy decreased from 87.0% to 59.3%, sensitivity from 96.3% to 77.8%, and specificity from 77.8% to 40.7%. False-negative and false-positive rates increased from 3.7% to 22.2% and from 22.2% to 59.3%, respectively. Observer confidence also decreased significantly (3.53 ± 0.90 vs. 2.61 ± 0.86; *p* < 0.001). No statistically significant differences in diagnostic accuracy were detected among the three edema-volume groups. **Conclusions:** Simulated soft-tissue edema substantially impairs ultrasonographic detection of nondisplaced dentoalveolar fractures. These experimental findings suggest that negative ultrasonographic findings obtained in the presence of soft-tissue swelling should be interpreted cautiously, with complementary imaging considered when clinically indicated.

## Simple Summary

Dentoalveolar fractures may be difficult to detect when post-traumatic soft-tissue swelling is present. Ultrasonography is a radiation-free and readily available imaging method, but the effect of swelling on its diagnostic performance is unclear. In this experimental study, simulated soft-tissue edema reduced the ability of ultrasonography to correctly identify nondisplaced dentoalveolar fractures and also decreased observer confidence. No significant differences in diagnostic accuracy were found among the three saline volumes used to simulate edema. These findings suggest that soft-tissue swelling may make subtle dentoalveolar fractures more difficult to assess with ultrasonography and that negative or uncertain findings should be interpreted cautiously.

## 1. Introduction

Dentoalveolar fractures are among the most common traumatic injuries affecting the oral and maxillofacial region, accounting for a substantial proportion of facial trauma, particularly in children and young adults [1,2]. Although these injuries are rarely life-threatening, they are associated with significant long-term dental morbidity; delayed or missed diagnosis may result in tooth loss, occlusal disturbances, periodontal complications, and impaired oral function [3]. Accurate diagnosis is therefore essential for appropriate treatment planning and favorable patient outcomes.

Clinical examination represents the initial step in diagnosing dentoalveolar fractures; however, its diagnostic accuracy is frequently compromised by pain, hemorrhage, limited mouth opening, and particularly post-traumatic soft-tissue swelling [3,4]. Conventional radiographic techniques, including periapical and panoramic radiography, remain the traditional first-line tools, yet they suffer from inherent limitations in identifying subtle or nondisplaced fractures due to anatomical superimposition and their two-dimensional nature [5]. Cone-beam computed tomography (CBCT) provides superior visualization of dentoalveolar structures and serves as the reference standard for complex trauma; nevertheless, its higher radiation dose, increased cost, and limited availability restrict its routine application in all emergency cases [5,6].

Ultrasonography has emerged as a promising alternative for evaluating maxillofacial injuries because it is non-invasive, free of ionizing radiation, portable, cost-effective, and capable of providing real-time imaging [2,7]. High-frequency linear transducers allow direct visualization of the cortical bone surface as a continuous hyperechoic line, whereas fractures appear as interruptions of this reflective interface [8,9]. Recent technological advances, including high-frequency portable systems, have further expanded the role of ultrasonography in point-of-care assessment [10,11]. Growing evidence has demonstrated high diagnostic accuracy for fractures involving the zygomatic arch, mandible, nasal bones, and orbital region, supporting its value as an adjunctive modality in selected trauma settings [7,11,12,13,14,15].

Despite these encouraging findings, evidence regarding dentoalveolar fractures remains limited, and most published studies have evaluated ultrasonography under routine clinical conditions without specifically isolating the influence of post-traumatic soft-tissue edema [16]. Because edema develops rapidly after facial trauma, it may substantially alter ultrasonographic image quality. Increased soft tissue thickness attenuates ultrasound transmission and may reduce the delineation of subtle cortical discontinuities. However, the extent to which post-traumatic soft-tissue edema affects ultrasonographic fracture detection remains unclear.

To the best of our knowledge, no previous study has specifically investigated the effect of soft-tissue edema on the diagnostic performance of ultrasonography for dentoalveolar fracture detection. Therefore, the present study aimed to determine the diagnostic performance of high-frequency ultrasonography against a CBCT reference standard for detecting nondisplaced dentoalveolar fractures and, more importantly, to isolate the effect of simulated post-traumatic soft-tissue edema. Clarifying this effect may help determine how soft-tissue swelling should be considered when interpreting ultrasonographic findings in the diagnostic assessment of dentoalveolar trauma. We hypothesized that simulated edema would significantly reduce ultrasonographic detection of the subtle cortical discontinuities associated with nondisplaced dentoalveolar fractures.

## 2. Materials and Methods

This experimental, ex vivo, single-blinded diagnostic accuracy study evaluated high-frequency ultrasonography against a CBCT reference standard for detecting nondisplaced dentoalveolar fractures and investigated the influence of simulated post-traumatic soft-tissue edema on fracture detection. The CBCT reference standard was established by an independent oral and maxillofacial radiologist who did not participate in the observers’ ultrasonographic evaluations.

### 2.1. Specimens

A total of 18 fresh sheep heads obtained from animals bred for human consumption were used. Six anatomically distinct dentoalveolar regions were evaluated in each specimen: the anterior maxilla, right posterior maxilla, left posterior maxilla, anterior mandible, right posterior mandible, and left posterior mandible. This yielded 108 evaluation sites, of which 54 were assigned to the fracture condition and 54 served as non-fractured controls. Sheep heads were selected because the ovine model has been widely used in dental research and provides relevant anatomical characteristics for experimental investigations involving dentoalveolar structures [17]. A computer-generated randomization sequence was used to allocate the 108 anatomical sites to the fracture and control groups (54 sites each), while maintaining balanced representation across the six predefined anatomical regions: anterior maxilla, right posterior maxilla (premolar–molar region), left posterior maxilla (premolar–molar region), anterior mandible, right posterior mandible (premolar–molar region), and left posterior mandible (premolar–molar region). The specimens were obtained post mortem from animals bred and slaughtered for human consumption; no animals were sacrificed specifically for this study.

### 2.2. Sample Size Estimation

An a priori sample size calculation was performed using G*Power software (version 3.1.9.7; Düsseldorf, Germany). Based on a chi-square goodness-of-fit test with a medium effect size (Cohen’s w = 0.30), an alpha level of 0.05, 80% statistical power, and 2 degrees of freedom, the minimum required sample size was estimated as 108 anatomically distinct evaluation sites. Accordingly, 108 sites from 18 sheep heads were included. Each site was independently assessed by two blinded observers, yielding 216 observer-level evaluations.

**Figure 1:**
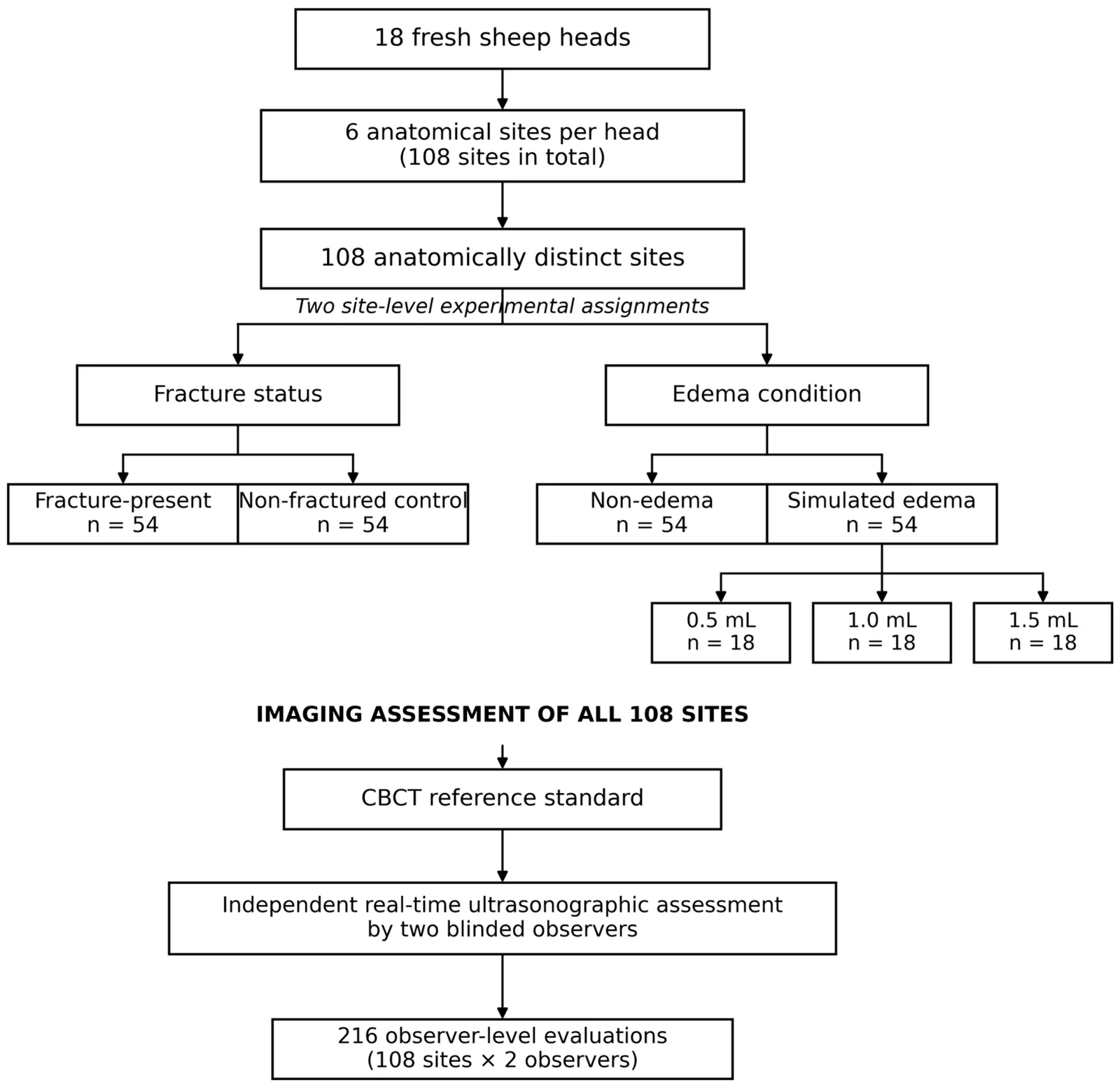
Flow diagram of the experimental design, site-level fracture and edema assignments, and imaging assessments.

The anatomical site was considered the experimental unit because fracture status, edema condition, CBCT reference classification, and ultrasonographic assessment were defined at the site level. The sample-size calculation was therefore performed at the anatomical-site level and did not incorporate an additional design effect for multiple anatomical sites originating from the same sheep head. A flow diagram summarizing specimen allocation, experimental conditions, and ultrasonographic assessments is provided in Figure 1.

### 2.3. Fracture Simulation

Nondisplaced alveolar fractures were experimentally induced by applying mechanical force directly to the alveolar bone using dental forceps. The forceps were applied to the alveolar bone, and force was gradually applied in the buccolingual direction. Force application was discontinued when a fracture was clinically suspected, with the aim of producing a fracture without intentional displacement. Fracture presence or absence was subsequently confirmed by CBCT, which served as the reference standard. A fracture was considered nondisplaced when a fracture line was identifiable on CBCT without visible separation, step-off, or displacement of the adjacent alveolar bone fragments. No quantitative displacement threshold was used.

### 2.4. Edema Simulation

**Figure 2:**
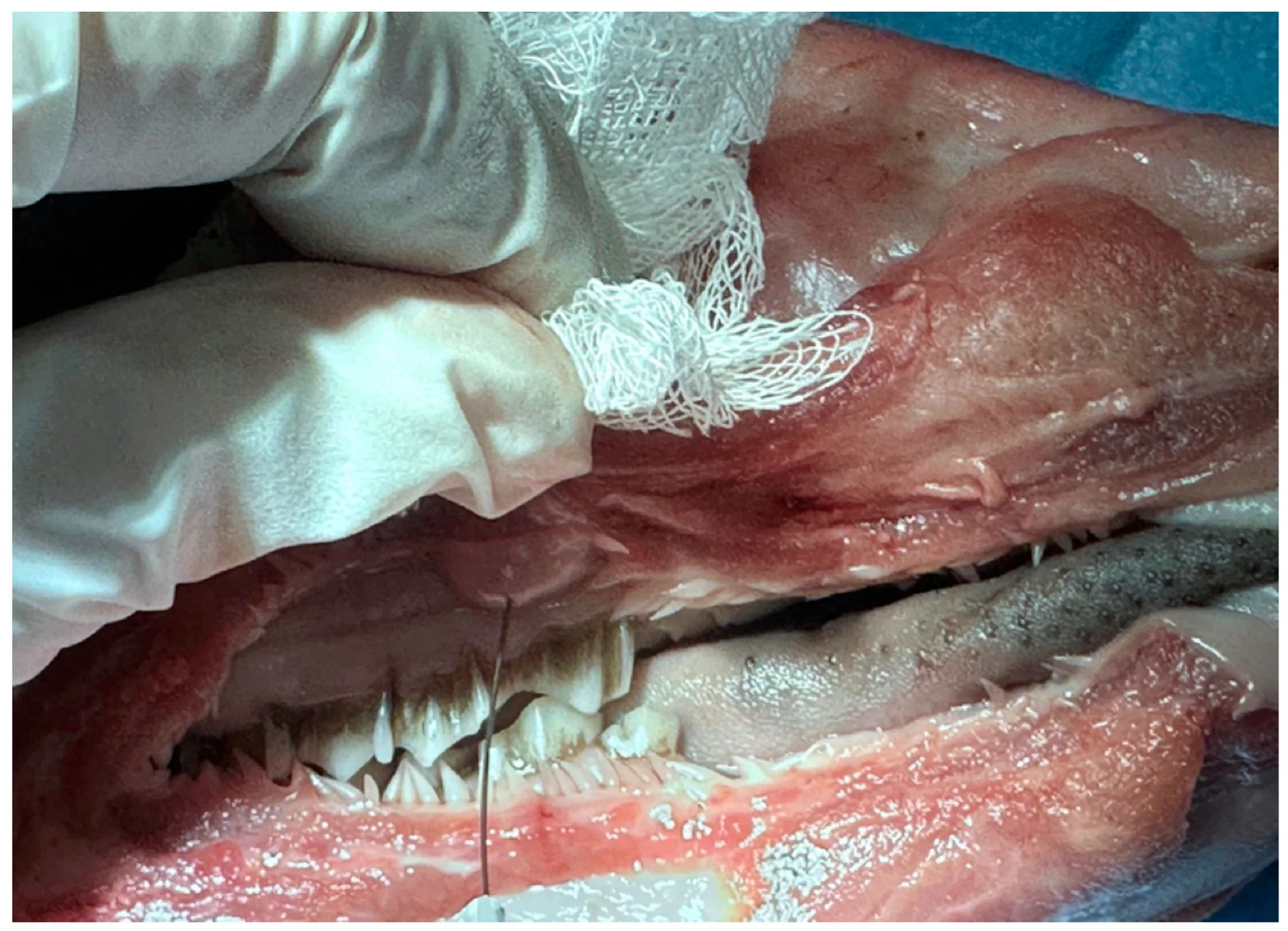
Representative photograph of the experimental simulation of localized soft-tissue edema. Saline was injected submucosally over the predetermined dentoalveolar evaluation site to produce localized swelling.

After fracture induction, simulated post-traumatic soft-tissue edema was created by submucosal injection of sterile normal saline over the predetermined evaluation site (Figure 2). This technique was selected to reproduce localized swelling encountered in early post-traumatic assessment while allowing standardized evaluation of edema as an isolated experimental factor.

Three injection volumes (0.5, 1.0, and 1.5 mL) were selected to provide standardized, progressively increasing experimental saline-volume conditions rather than to represent established clinical edema-severity categories. These volumes were not based on established clinical thresholds or preliminary soft-tissue thickness measurements. Post-injection soft-tissue thickness was not quantitatively measured by ultrasonography; therefore, injected saline volume was used as the experimental indicator of simulated edema level. The injected saline remained localized within the submucosal tissues during ultrasonographic examination without visible leakage. Edema volume was assigned to each evaluation site using a computer-generated randomization sequence before image acquisition. The non-edema and edema conditions comprised different anatomical sites; the same site was not evaluated both before and after edema induction. Of the 108 anatomical sites, 54 were assigned to the non-edema condition and 54 to the edema condition. Each site was independently evaluated by two observers, resulting in 108 observer-level evaluations per edema condition and 216 observer-level evaluations overall. To minimize observer expectation bias and avoid carryover effects, each site received only one randomly assigned edema volume; sequential injections were not performed at the same site.

Ultrasonographic examinations were then performed under standardized imaging conditions. Edema formation was verified by the presence of localized soft-tissue swelling at the injection site without visible saline leakage. The extent of simulated edema was controlled by administering predefined saline volumes (0.5, 1.0, or 1.5 mL) using the same injection approach across sites.

### 2.5. CBCT Acquisition

Imaging was performed using a Scanora^®^ 3D-X CBCT unit (Soredex, Tuusula, Finland). Specimens were positioned upright and stabilized. Head positioning was standardized by aligning the midsagittal plane perpendicular to the floor and confirming alignment on scout views before exposure. Scans were acquired using an 8 × 16 cm field of view and a 0.2 mm isotropic voxel size. A fracture was defined as a visible cortical discontinuity on multiplanar CBCT reconstructions, as determined by the reference examiner.

### 2.6. Ultrasonographic Examination

**Figure 3:**
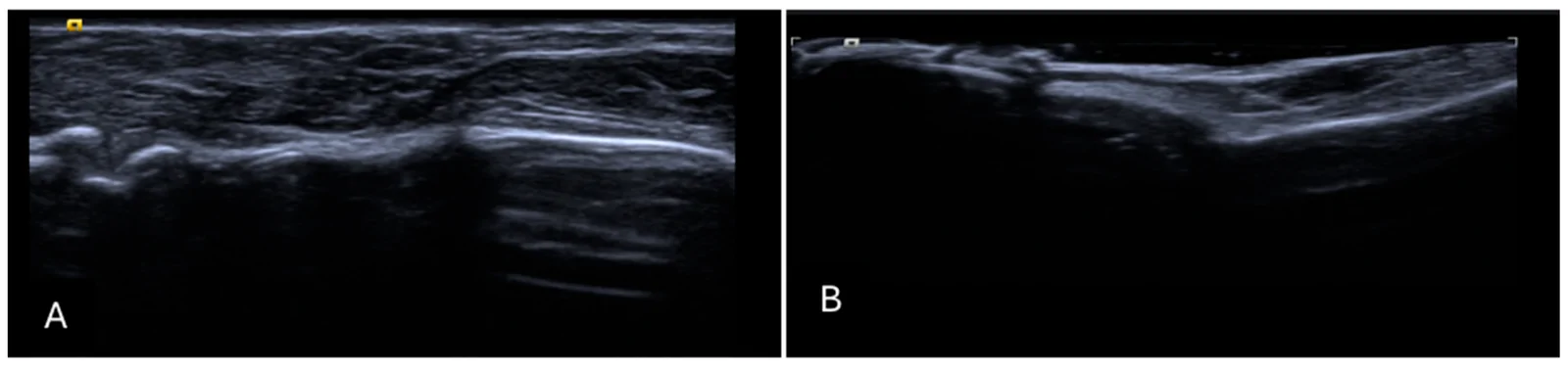
Representative ultrasonographic images of a nondisplaced dentoalveolar fracture under simulated edematous and non-edematous conditions. (**A**) In the presence of simulated soft-tissue edema, the cortical surface and fracture-related discontinuity are less conspicuous. (**B**) In the absence of simulated edema, the cortical discontinuity associated with the fracture is more clearly visualized.

Ultrasonographic examinations were performed using an ACUSON Juniper system (Siemens Healthineers, Erlangen, Germany) equipped with an 18H5 high-frequency linear transducer. Examinations used the intraoral preset, tissue harmonic imaging at 10.6 MHz, and an imaging depth of approximately 3 cm. Overall gain was adjusted between 0 and 20 dB when required to optimize cortical visualization; all other imaging parameters were kept constant. Each site was examined in real time and independently by both observers in separate sessions. Each observer performed probe positioning and image acquisition and recorded findings independently; shared static images or cine loops were not used for interpretation. Ultrasound gel was applied to ensure adequate acoustic coupling. The transducer was positioned over the buccal soft tissues corresponding to each predetermined site, with minimal probe pressure to avoid compressing the simulated edema. Cortical integrity was assessed from the continuity of the hyperechoic cortical surface; any focal interruption of this line was recorded as a fracture (Figure 3). After each examination, observers recorded fracture presence or absence and rated confidence on a five-point Likert scale (1, not confident at all; 2, slightly confident; 3, moderately confident; 4, confident; 5, very confident).

### 2.7. Observer Characteristics

Two oral and maxillofacial radiologists (S.A.T. and M.M.A.) participated as blinded observers.

Three weeks before the experimental assessments, the observers underwent a calibration session using 12 separate evaluation sites that were not included in the study sample. Agreement in the calibration dataset was substantial (Cohen’s κ = 0.749; 95% CI, 0.392–0.905; *p* = 0.002). After calibration, the observers independently evaluated the study sites while blinded to fracture allocation, simulated edema volume, and each other’s assessments. Saline injections were performed before the ultrasonographic examinations without the observers witnessing the injection procedure, and the injected volume (0.5, 1.0, or 1.5 mL) was not disclosed to them. Although the presence of localized swelling could be apparent during real-time examination, the observers remained blinded to the amount of saline administered.

### 2.8. Statistical Analysis

Descriptive statistics were reported as frequency (*n*), percentage (%), mean ± standard deviation (SD), median, minimum, and maximum, as appropriate. Normality of continuous variables was assessed using the Shapiro–Wilk test. Diagnostic performance was determined by comparing ultrasonographic classifications with the CBCT reference standard. Sensitivity, specificity, overall accuracy, false-negative rate, and false-positive rate were calculated with corresponding 95% confidence intervals. To account for the non-independence of the two observer assessments obtained from each anatomical site, the association between simulated edema and correct ultrasonographic classification was analyzed using a binary logistic generalized estimating equation (GEE) model, with anatomical site specified as the clustering variable and observer included as a fixed effect. Odds ratios (ORs) with 95% confidence intervals were reported. Associations between categorical variables were evaluated using Pearson’s chi-square test when expected cell frequencies exceeded 5, and effect size was expressed as Cramer’s V. Observer confidence scores were compared between edema-present and edema-absent conditions using the Mann–Whitney U test, with effect size calculated as r = |Z|/√N. Interobserver agreement in the pre-study calibration exercise was assessed using Cohen’s kappa with 95% confidence intervals. Analyses were performed in IBM SPSS Statistics for Windows, version 27.0 (IBM Corp., Armonk, NY, USA). All tests were two-sided, and *p* < 0.05 was considered statistically significant.

## 3. Results

### 3.1. Diagnostic Performance According to Simulated Edema Presence

In the binary logistic GEE model accounting for clustering of observer assessments within anatomical sites, simulated soft-tissue edema was associated with significantly lower odds of correct ultrasonographic classification (OR = 0.217; 95% CI, 0.096–0.491; *p* < 0.001). Observer was not significantly associated with classification accuracy (OR = 1.23; 95% CI, 0.75–2.04; *p* = 0.413). In the absence of edema, ultrasonography demonstrated a sensitivity of 96.3% (95% CI, 87.5–99.0), a specificity of 77.8% (95% CI, 65.1–86.8), and an overall accuracy of 87.0% (95% CI, 79.4–92.1). In the presence of simulated edema, sensitivity decreased to 77.8% (95% CI, 65.1–86.8), specificity to 40.7% (95% CI, 28.7–54.0), and overall accuracy to 59.3% (95% CI, 49.8–68.1). The false-negative rate increased from 3.7% to 22.2%, whereas the false-positive rate increased from 22.2% to 59.3%, indicating that simulated edema adversely affected both fracture detection and the correct exclusion of fracture, with a more pronounced reduction in specificity (Table 1).

### 3.2. Diagnostic Performance According to Simulated Edema Volume

**Figure 4:**
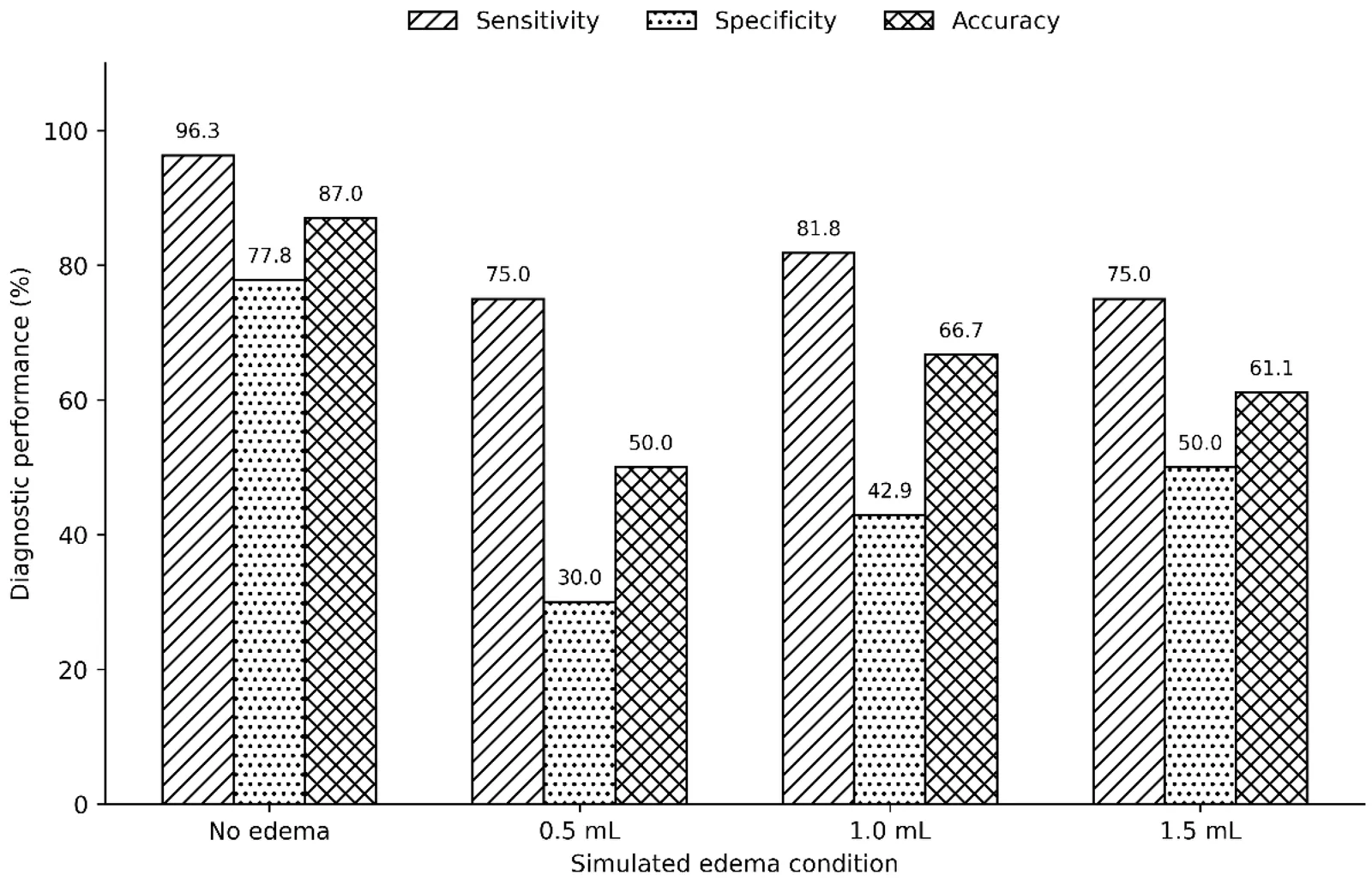
Sensitivity, specificity, and overall diagnostic accuracy of ultrasonography according to simulated edema condition and injected saline volume. Values are presented as percentages.

Overall diagnostic accuracy differed significantly across the four edema-volume conditions (Pearson’s χ^2^ = 23.853, *p* < 0.001, Cramer’s V = 0.332). Accuracy was highest in the absence of edema (87.0%) and decreased to 50.0%, 66.7%, and 61.1% in the 0.5, 1.0, and 1.5 mL edema groups, respectively. Bonferroni-adjusted post hoc comparisons demonstrated that diagnostic accuracy in the non-edema condition was significantly higher than that in each of the three edema-volume groups, whereas no statistically significant differences were observed among the 0.5, 1.0, and 1.5 mL groups. Sensitivity was 96.3% in the non-edema condition and ranged from 75.0% to 81.8% across the edema groups, while specificity decreased from 77.8% in the absence of edema to 30.0–50.0% in the edema groups. No monotonic dose–response pattern was observed with increasing edema volume (Table 2). The diagnostic performance measures across the four edema conditions are additionally illustrated in Figure 4.

### 3.3. Observer Confidence Scores

Observer confidence scores were significantly lower in the presence of simulated soft-tissue edema than in its absence (Table 3). The mean confidence score decreased from 3.53 ± 0.90 (median, 4) in the non-edema condition to 2.61 ± 0.86 (median, 3) in the edema condition (Mann–Whitney U test, Z = −7.200, *p* < 0.001; effect size, r = 0.490). These findings indicate that simulated soft-tissue edema significantly reduced the observers’ confidence in their ultrasonographic assessments.

## 4. Discussion

To our knowledge, this is the first controlled ex vivo study specifically designed to isolate the effect of simulated post-traumatic soft-tissue edema on ultrasonographic detection of nondisplaced dentoalveolar fractures. Edema significantly reduced sensitivity, specificity, overall accuracy, and observer confidence, while increasing both false-negative and false-positive findings. No statistically significant differences in diagnostic accuracy were detected among the 0.5, 1.0, and 1.5 mL groups. However, given the limited number of assessments within each edema-volume subgroup, this finding should not be interpreted as evidence that edema volume has no effect on diagnostic performance.

The reduction in diagnostic performance observed in the presence of simulated edema is biologically and physically plausible. Ultrasonographic identification of fractures depends on clear visualization of the hyperechoic cortical bone surface, with fractures recognized as focal interruptions or irregularities of this highly reflective interface [9,16]. Because nondisplaced dentoalveolar fractures produce only subtle cortical discontinuities, their detection is particularly dependent on high image quality and spatial resolution [16]. In the present study, simulated edema altered the overlying soft-tissue layer between the ultrasound transducer and the cortical surface. This additional tissue layer may have altered ultrasound propagation and reduced the conspicuity of the cortical interface, making subtle cortical interruptions more difficult to appreciate. Because fracture identification relies on detecting a small focal interruption in an otherwise continuous hyperechoic cortical line, reduced conspicuity of the cortical interface may preferentially obscure these subtle discontinuities even when the overall ultrasonographic image remains interpretable. Rather than reflecting a direct effect of the saline itself, these findings are more likely attributable to changes in the acoustic pathway and the increased separation between the transducer and the cortical surface, both of which may reduce the visibility of fine cortical irregularities [9,16,18].

Our findings are consistent with previous investigations demonstrating that alterations in the overlying soft tissues can influence ultrasonographic assessment. Johansen and Stone [19] reported that peripheral soft-tissue edema significantly affected quantitative bone ultrasound measurements by modifying ultrasound propagation through the intervening tissues. Although their study focused on bone densitometry rather than fracture detection, it supports the concept that edema itself can influence ultrasound-derived information independently of the underlying osseous condition. Similarly, Zhang et al. [18] demonstrated that increasing subcutaneous edema affects ultrasonographic assessment of superficial tissues, emphasizing that soft tissue changes should be considered an important determinant of image quality. While neither study specifically evaluated maxillofacial fractures, together they provide a plausible explanation for the significant reduction in diagnostic performance and observer confidence observed in the present study.

An additional finding of the present study was that no statistically significant differences in diagnostic accuracy were detected among the three injected saline-volume groups, and no clear monotonic pattern was observed. However, injected saline volume should not be interpreted as a direct quantitative measure of edema severity because post-injection soft-tissue thickness was not measured. Furthermore, the limited number of sites within each volume subgroup and the relatively narrow range of injected volumes may have reduced the ability to detect a dose–response relationship. Although the lack of significant differences among the three saline-volume groups could be compatible with a threshold-like effect, this interpretation remains speculative because post-injection soft-tissue thickness was not directly measured and the subgroup sample sizes were limited. Therefore, the present findings do not establish either the presence or absence of a volume-dependent effect, and this relationship should be investigated in future studies using direct quantitative measurements of soft-tissue thickness and larger samples.

Previous clinical studies have generally reported high diagnostic performance for ultrasonography in maxillofacial fracture detection, although considerable variation has been observed according to fracture location and study design [4,10,11,12,15]. A meta-analysis reported pooled sensitivity and specificity of 94% and 96%, respectively, while individual clinical studies have demonstrated particularly high performance for superficial fractures involving the zygomatic arch and mandible [10,11,12,15]. These values are generally closer to, or even higher than, those observed in the non-edema condition of the present study than in the simulated-edema condition. Therefore, the current findings should not be interpreted as indicating that post-traumatic edema alone explains the variability reported in previous clinical studies. Differences in fracture location, displacement, soft-tissue anatomy, transducer characteristics, operator experience, and reference standards may also substantially influence diagnostic performance. Rather, our controlled experimental findings identify soft-tissue edema as one potential factor capable of reducing ultrasonographic performance, whose relative contribution in clinical trauma populations remains to be established.

Evidence from other anatomical regions also illustrates the complex relationship between soft-tissue changes and ultrasonographic fracture assessment. In calcaneal stress fractures, Bianchi and Luong reported subcutaneous edema and periosteal thickening in all examined patients, together with cortical irregularity in most cases and periosteal hypervascularity in all cases [20]. Similarly, a systematic review of lower-extremity stress fractures identified periosteal thickening, cortical disruption or irregularity, and periosteal and soft-tissue hypervascularity as recurring ultrasonographic findings [21]. These observations suggest that fracture-associated soft-tissue changes may coexist with, and in some settings provide indirect signs during, ultrasonographic fracture assessment rather than uniformly impairing it. In contrast, the present study specifically isolated simulated edema as an experimental factor and demonstrated reduced detection of subtle cortical discontinuities in nondisplaced dentoalveolar fractures. This distinction may reflect differences in anatomical region, fracture characteristics, and the sonographic features used for fracture identification.

A similar concept has been proposed by Al-Ani et al. [22], who recommended repeating ultrasonographic examination after the resolution of soft tissue swelling when clinical suspicion persists despite an initially inconclusive examination. Although their study on nasal fractures did not demonstrate a statistically significant improvement after edema resolution, our controlled experimental model supports the biological plausibility of this recommendation by demonstrating that edema alone can significantly reduce ultrasonographic diagnostic performance. Together, these findings suggest that the timing of ultrasonographic examination may influence its diagnostic reliability in patients presenting with acute maxillofacial trauma.

An important secondary finding was the significant reduction in observer confidence in the presence of simulated edema. This decrease closely paralleled the reduction in diagnostic performance, suggesting that soft tissue swelling affects not only the objective ability to identify cortical disruptions but also the examiner’s subjective certainty during image interpretation. Such concordance between reduced confidence and reduced diagnostic accuracy may have practical clinical relevance, as diminished observer confidence could appropriately prompt further radiographic evaluation when ultrasonographic findings are equivocal.

From a clinical perspective, the present findings reinforce the role of ultrasonography as a valuable adjunct rather than a replacement for conventional imaging in the assessment of dentoalveolar trauma. Ultrasonography remains particularly attractive because it is radiation-free, readily accessible, portable, and capable of providing real-time assessment of superficial maxillofacial structures [10,16]. Our findings are also consistent with those of Johansen and Stone [19], who demonstrated that edema itself may influence ultrasound-derived measurements by altering sound propagation through the overlying soft tissues, supporting the concept that soft tissue swelling should be regarded as an important determinant of ultrasonographic performance. Nevertheless, the present results indicate that simulated post-traumatic soft-tissue edema may substantially reduce both the sensitivity and specificity of ultrasonographic fracture detection. Consequently, negative ultrasonographic findings obtained in the presence of pronounced soft tissue swelling should be interpreted with caution, particularly when clinical suspicion for a nondisplaced dentoalveolar fracture remains high. Under such circumstances, complementary imaging, such as CBCT, or close clinical follow-up may be considered when clinically justified, particularly when suspicion for fracture persists.

Several limitations should be considered. First, this ex vivo ovine model cannot fully reproduce the biological and clinical complexity of human dentoalveolar trauma. Second, submucosal saline injection provided a standardized model of localized swelling but did not reproduce inflammation, hemorrhage, tissue heterogeneity, pain, or patient movement. Furthermore, post-injection soft-tissue thickness was not quantitatively measured; therefore, the administered saline volume should not be interpreted as a direct measure of edema severity. Accordingly, the magnitude of the observed effect of simulated edema may not directly reflect that encountered in clinical trauma and may overestimate its impact on ultrasonographic diagnostic performance in vivo. Third, only nondisplaced dentoalveolar fractures were evaluated using one ultrasound system and two experienced oral and maxillofacial radiologists, which may limit generalizability to other fracture types, equipment, and operator experience levels. Fourth, the range of simulated edema volumes was relatively narrow, and each edema-volume subgroup contained a limited number of assessments. Finally, although within-site clustering of the two observer assessments was accounted for using GEE, multiple anatomical sites originated from the same sheep head, which should be considered when interpreting the precision of the estimates.

## 5. Conclusions

Simulated post-traumatic soft-tissue edema significantly impaired ultrasonographic detection of nondisplaced dentoalveolar fractures, reducing sensitivity, specificity, overall accuracy, and observer confidence while increasing false-negative and false-positive findings. Ultrasonography remains a useful radiation-free adjunct for superficial dentoalveolar assessment, but findings obtained in the presence of soft-tissue swelling should be interpreted cautiously. These experimental findings suggest that, in a clinical setting, persistent suspicion despite a negative or equivocal ultrasonographic examination may warrant consideration of complementary imaging, such as CBCT, when clinically justified. Future studies should validate these findings in clinical populations using direct quantitative assessment of soft-tissue thickness, larger sample sizes, and different ultrasonographic systems and operators.

## Figures and Tables

**Table 1:** Diagnostic performance of ultrasonography according to the presence of simulated soft-tissue edema.

Edema Condition	TP	FN	FP	TN	Sensitivity, % (95% CI)	Specificity, % (95% CI)	Accuracy, % (95% CI)
**Absent**	52	2	12	42	96.3 (87.5–99.0)	77.8 (65.1–86.8)	87.0 (79.4–92.1)
**Present**	42	12	32	22	77.8 (65.1–86.8)	40.7 (28.7–54.0)	59.3 (49.8–68.1)

Notes: Values are based on observer-level assessments. Each edema condition included 54 distinct anatomical sites evaluated independently by two observers (108 assessments per condition). TP, true positive; FN, false negative; FP, false positive; TN, true negative; CI, confidence interval. The association between simulated edema and correct ultrasonographic classification was evaluated using a binary logistic generalized estimating equation (GEE) model accounting for clustering of observer assessments within anatomical sites, with observer included as a fixed effect.

**Table 2:** Diagnostic performance of ultrasonography according to simulated edema volume.

Edema Volume	TP	FN	FP	TN	Sensitivity, % (95% CI)	Specificity, % (95% CI)	Accuracy, % (95% CI)
**None**	52	2	12	42	96.3 (87.5–99.0)	77.8 (65.1–86.8)	87.0 (79.4–92.1)
**0.5 mL**	12	4	14	6	75.0 (50.5–89.8)	30.0 (14.5–51.9)	50.0 (34.5–65.5)
**1.0 mL**	18	4	8	6	81.8 (61.5–92.7)	42.9 (21.4–67.4)	66.7 (50.3–79.8)
**1.5 mL**	12	4	10	10	75.0 (50.5–89.8)	50.0 (29.9–70.1)	61.1 (44.9–75.2)

Notes: Values are based on observer-level assessments. The non-edema condition comprised 108 assessments, and each edema-volume group comprised 36 assessments. TP, true positive; FN, false negative; FP, false positive; TN, true negative. Overall differences in diagnostic accuracy were significant (Pearson’s χ^2^ = 23.853, *p* < 0.001; Cramer’s V = 0.332), followed by Bonferroni-adjusted pairwise comparisons. Sensitivity, specificity, and accuracy are presented with 95% confidence intervals (CIs). Sensitivity and specificity are presented descriptively.

**Table 3:** Observer confidence scores according to simulated edema condition.

Edema Presence	Min–Max	Mean ± SD (Median)	Test Statistic	*p*	Effect Size
**Absent**	1–5	3.53 ± 0.90 (4)			
**Present**	1–5	2.61 ± 0.86 (3)	Z = −7.200	<0.001	r = 0.490

Notes: Values are presented as mean ± standard deviation (SD), median, and range. Observer confidence was rated using a five-point Likert scale: 1, not confident at all; 2, slightly confident; 3, moderately confident; 4, confident; and 5, very confident. Comparisons between the edema-present and edema-absent conditions were performed using the Mann–Whitney U test. Effect size was calculated as r = |Z|/√N. A *p*-value <0.05 was considered statistically significant.

## Data Availability

The raw data supporting the conclusions of this article will be made available by the authors on request.
